# Transfer of Pesticide Residues from Grapes (*Vitis vinifera*) into Wine—Correlation with Selected Physicochemical Properties of the Active Substances

**DOI:** 10.3390/toxics10050248

**Published:** 2022-05-16

**Authors:** Arno Kittelmann, Carola Müller, Sascha Rohn, Britta Michalski

**Affiliations:** 1 Department Pesticides Safety, German Federal Institute for Risk Assessment, 10589 Berlin, Germany; britta.michalski@bfr.bund.de; 2 Department of Life Science and Technology, Berliner Hochschule für Technik, 13353 Berlin, Germany; cmueller@bht-berlin.de; 3 Department of Food Chemistry and Analysis, Institute of Food Technology and Food Chemistry, Technische Universität Berlin, 13355 Berlin, Germany; rohn@tu-berlin.de

**Keywords:** pesticide, residues, processing factor, winemaking, wine

## Abstract

The concentration of pesticide residues in agricultural products at harvest can change during subsequent processing steps. This change, commonly expressed as Processing Factor (PF), is influenced by the raw agricultural commodity, and the processing conditions, as well as the properties of the substances. As it is not possible to conduct processing studies for all possible combinations of pesticide × process × product, new approaches for determining processing factors are needed. Wine was chosen as the object of the present study because it is a widely consumed product. Furthermore, it is already known that the concentration of pesticide residues can change considerably during the processing of grapes into wine, substantiating the need for PFs for a large number of pesticides. The aim of the present study was to investigate the correlation between selected physicochemical properties and PFs. In addition, the influence of different winemaking processes was explored. For this purpose, 70 processing studies conducted by pesticide manufacturers in the framework of regulatory procedures were evaluated in detail and PFs were derived for 20 pesticides. For wine, a good correlation between the PF and the octanol-water partition coefficient of the substances was found, depending on the specific production methods used. Exemplarily, the coefficient of determination for white wine was 0.85, and 0.81 for red wine, when thermovinification was applied. These results can serve as the basis for a predictive model to be validated further with future winemaking studies for pesticides.

## 1. Introduction

Knowledge of changes in pesticide residues during food processing is essential for a realistic estimate of consumer exposure and risk, as most of the foods are consumed in a processed form. Also, food control authorities make use of processing information: it allows them to recalculate the pesticide concentration found in a processed product to the respective concentration in the raw product, to which maximum residue levels (MRLs) apply [1].

The so-called Processing factor (PF) is calculated from the ratio of the pesticide residue concentration in the raw product to the pesticide residue concentration in the processed product, as shown in Equation (1).

A PF higher than 1 reflects an enrichment of pesticide residues in the processed product, whereas a factor below 1 indicates a decrease of residues. It is specific to the pesticide, to the raw material, and to the processed food being produced from it. Due to the high number of possible combinations, it is impossible to cover all of them in experiments.

(1) $$Processing\;factor=\frac{Pesticide\;residue\;level\;in\;processed\;product}{Pesticide\;residue\;level\;in\;raw\;product}$$

To study transfer processes of pesticides, winemaking is a reasonable example for the following reasons: (i) it is an important food processing operation, (ii) it is already known to alter residues considerably, and (iii) it is important with regard to the amount of wine consumed worldwide [2,3,4].

The European Union is the world’s largest wine producer with a production volume of around 156 million hectoliters per year [5]. To protect grapevines (*Vitis vinifera* L.) from competing weeds, bacterial and viral diseases, fungi, insects, and further pests, they are commonly treated with pesticides [6,7]. Depending on factors such as chemical structure and physicochemical properties of the substances, but also ecophysiology, and weather conditions (e.g., humidity, rainfall, temperature), as well as agronomic practices, pesticide residues sometimes remain in or on the grapes at harvest [8]. Measurable residues in grapes are particularly expected for persistent compounds being applied at high rates and frequencies during the vegetation period, and for compounds being applied shortly before harvest [9].

During the process of winemaking, pesticide residues are transferred from grapes into the final product, wine, to some extent, but usually the residue concentration in wine is much lower than in the original grapes [10]. Literature describes that the various technological sub-processes involved in winemaking, such as pressing, fermentation, fining, and filtration, generally lead to a reduction of the residues [11,12,13,14].

In a study with spiked samples, Pazzirota et al. showed a strong correlation of the change in pesticide content over the production process with the octanol-water coefficient of the substances [15].

Further specifics of the winemaking process can also influence the pesticide concentration in wine. For example, the impact of different fining agents on the level of pesticide residues has been investigated in various studies [16,17]. As fining agents contribute to the removal of solids, they can influence the transfer of pesticides into the wine, depending on the type and quantity used. Also, levels of pesticide residues can be affected by malolactic fermentation [18].

Studies on pesticide residue transfer during processing operations, such as winemaking, are often conducted with spiked samples, as they are easier to obtain and allow a large number of pesticides to be analyzed at once [2,13,14,15,16]. Untreated raw material (such as the grapes) is mixed (spiked) with a defined amount of one or more pesticides prior to processing. However, using spiked samples does not adequately reflect realistic conditions and is not accepted in the regulatory context [19,20]. *In praxi*, grapes are treated with pesticides during the whole vegetation period, allowing the active substances to dislocate or to metabolize within the plants or to interact with the plant tissues over certain periods of time, depending on the timing of application and harvest [21,22,23].

In the present study, grape processing studies were evaluated, which have been submitted by pesticide manufacturers in support of pesticide authorization and approval, as well as MRL setting procedures. According to the European law, responsibility for conducting all required studies lies with the applicant, which is the pesticide manufacturer in most cases [1]. The manufacturer must be committed to good laboratory practice as well as to the provisions of OECD Test Guidelines. To mimic realistic conditions as closely as possible, guideline compliant processing studies have to be conducted with field-treated raw material, i.e., with agricultural products having been subject to a real pesticide treatment regime in the vegetation period in order to reflect all possible influences on the residue level [19].

The aim of the present study was to investigate the potential correlation of the physicochemical properties of pesticides and changes of the pesticides’ content during transformation from grapes to wine. This opens a way to predict processing factors for pesticides for which no corresponding processing studies are available.

## 2. Materials and Methods

### 2.1. Selection of Processing Studies

Studies on changes of pesticide residues during food processing are compiled in the “European database of processing factors for pesticides in food” [24,25]. This database contains information on more than 800 processing studies, conducted by pesticide manufacturers in compliance with OECD Test Guideline 508 [19]. All of them were conducted with raw material that had been treated with pesticides in the field according to common practice, leading to so called incurred residues. Those studies were submitted as part of the dossiers for EU active substance approval and MRL setting procedures. One hundred and sixty-six studies covered the processing of grapes (*Vitis vinifera*) into wine. Since the last update of the database, twenty additional processing studies on winemaking were submitted by pesticide manufacturers and also included in the data evaluation. Information obtained from these studies will be published in the next database update. As one of the authorities involved in pesticide approval, authorization and MRL setting procedures, the German Federal Institute for Risk Assessment (BfR), Berlin, Germany, has access to the full reports of all of these studies.

As for the EU database, the present work refers to PFs, according to the residue definition for monitoring. This residue definition denotes the components of the residue, which serve as a marker of the pesticide residue and for which MRLs are being established. As pesticide metabolites often have different physicochemical properties than their parent compounds, and can be present at variable levels, depending on the timing of treatment, the present work relies on those active substances, for which no metabolites are included in the residue definition, to minimize additional uncertainties [26]. Processing studies with residues in the final products being below the detection limit of the analytical method were disregarded, because PFs derived from such studies are only indicative and connected to large uncertainties.

A total of 70 studies were selected for this investigation and all of them were thoroughly re-evaluated with regards to the detailed processing conditions. PFs were extracted from the database and adapted when necessary. For example, in contrast to the database, values of younger wines were preferred over those for aged wines to minimize uncertainties which could be due to the effect of storage. A median PF was calculated for all pesticides for which sufficient data was available according to the provisions in OECD Guideline 508 [19]. This includes substances for which at least three PFs are available for the same process and processed product (must, white wine, or red wine) or for which two PFs were available, not deviating from each other by more than 50%. Twenty pesticides with a sufficiently broad database were identified and included in the study.

### 2.2. Selection of Physicochemical Properties

The winemaking process consists of the following essential steps: separation of the juice from the grape skins, seeds, and pulp and subsequent fermentation of the juice. Therefore, it can be assumed that such physicochemical properties of the pesticides are of particular importance, which affect their partition between the solid and the liquid phase. For the present study, the octanol-water partition coefficient (K_OW_) and the water solubility (S_W_) were hypothesized and selected as the most important parameters influencing the phase distribution of the pesticides. A study on apple juice already showed a correlation between log K_OW_ and PFs [27].

The log K_OW_ values and water solubilities of the 20 pesticides investigated in the present study are listed in Table 1. This information was taken from Assessment Reports prepared by the EU Member States and from Conclusions on pesticide peer reviews, as published by the EFSA, in the context of the active substance approval, and particularly from the so called List of Endpoints for each active substance, which is part of those publications. The pH value of wine is usually between 3.0 and 3.7 [28]. Therefore, data on water solubility and log K_OW_ value were primarily selected for acidic conditions (pH 3 to pH 5). For some of the investigated pesticides, no pH dependence was expected and no data was available for acidic pH values. In these cases, data for pH 7 were used.

### 2.3. Winemaking Process

In the studies taken into account, grapes were processed into wine according to common practice. For white wine, the grapes were crushed first. The juice was pressed off immediately, or after one to two hours. In some procedures, the mash was treated with pectolytic enzymes before pressing to increase the juice yield. After addition of the yeast, the must was fermented and filtered afterwards. In about half of the studies, the wine was clarified in addition to filtration, either by using bentonite or gelatin.

**Figure 1:**
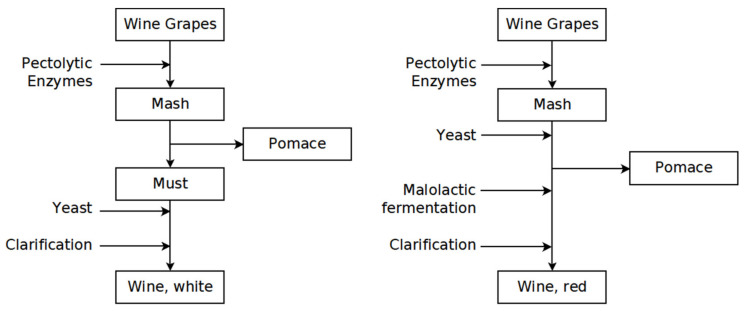
Flowcharts of the processes used in the investigated studies to prepare white wine and red wine (mash fermentation). The red wine production with thermovinification was analogous to the white wine production, with the only difference that the mash was heated to over 60 °C before pressing.

For red wine, two different methods were mainly used in the studies taken into account. In some studies, a classic must fermentation was carried out. In this process, the must was fermented, after crushing the grapes. The pomace was separated from the wine after fermentation. In the remaining studies, the thermovinification method was used, involving heating of the must to a temperature between 60 °C and 80 °C for 2–3 min. The pomace was then pressed and the resulting juice subjected to fermentation, similar to white wine. In contrast to white wine, almost all red wines were clarified with the help of fining agents, except for four studies. The main steps of white and red wine production are depicted in Figure 1.

Regulatory processing studies usually aim at approximating the most common conditions in winemaking practice. However, given the large variation in winemaking techniques, only limited sets of processing conditions can be covered by these experimental studies. For example, EU Regulation 2019/934 allows a much wider range of fining agents for winemaking than those used in the studies examined [29].

### 2.4. Statistical Analysis

Regression analysis was performed using GraphPad Prism version 8.2.0 for Windows, GraphPad Software, San Diego, CA, USA. Linear regression was used to estimate the correlation of the physicochemical properties of the pesticides and the extent of residue transfer from grapes into wine (characterized by the PF). As the most important physicochemical properties in this context, the water solubility and the octanol-water partition coefficient were hypothesized.

Multiple linear regression was used to additionally determine the influence of different processing methods. In addition to the above-mentioned physicochemical properties, it was investigated whether fining, the use of pectolytic enzymes, malolactic fermentation, and thermovinification had an influence on the PF.

## 3. Results and Discussion

### 3.1. White Wine

The selected processing studies were evaluated with regards to the amounts of pesticides transferred from the grapes into the wine. PFs were calculated for wine and, where possible, for must. Table 2 summarizes the median PFs for the active substances investigated in white wine and if fining agents were used in the process to clarify the wine. The processing of grapes into white wine resulted in a decrease of pesticide concentrations, except for imidacloprid and thiophanate-methyl. In most cases, a considerable fraction of the pesticide seems to already be removed during the solid-liquid separation at the beginning of the winemaking process. Consequently, the PF for must is often comparable to that for wine. This is especially valid for non-clarified wine.

Based on the two available processing studies, imidacloprid exhibits a PF clearly >1, in must as well as in wine. This might be explained by its physicochemical properties. Imidacloprid has the lowest log K_OW_ value and the highest water solubility of all active substances considered and, consequently, was not expected to accumulate in the solid fraction removed during processing, but rather to remain in the aqueous phase.

The lowest PF was obtained for benzovindiflupyr. Again, this is in line with the physicochemical properties of the substance. It has the highest log K_OW_ value of all investigated pesticides and a very low water solubility of 0.98 mg/L.

**Figure 2:**
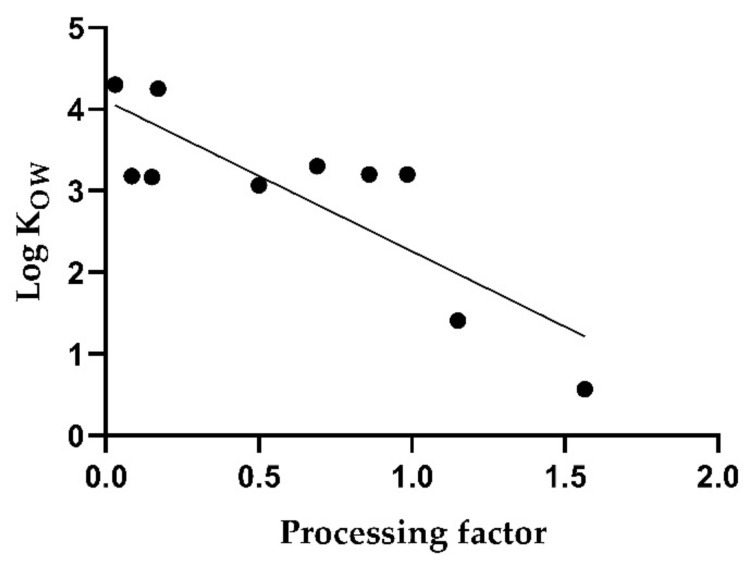
Correlation of processing factors with octanol-water partition coefficients of pesticide active substances for white wines. Each dot represents one of the pesticides in Table 2.

Although a correlation between the log K_OW_ and the PF can be observed, it is only moderate with a coefficient of determination of 0.70 (*p* < 0.005). Figure 2 shows that the PF varies, even for substances with similar log K_OW_ values, except for the most pronounced values. In the case of water solubility, correlation with the PF was insignificant (R^2^ = 0.36; *p* = 0.07).

In the second step of the evaluation, the influence of fining on the pesticide concentration in the end product wine was additionally considered. A multiple linear correlation was calculated, which took into account the log K_OW_ and whether fining was carried out or not. The equation obtained for processing factors for pesticides in white wine is as follows:

(2) $$\mathrm{PF}=1.919-0.37{\;\log\;K}_{\mathrm{OW}}-0.424\;C$$

with *C* indicating the fining process (*C* = 1 for fined wines and *C* = 0, when no fining was performed).

**Figure 3:**
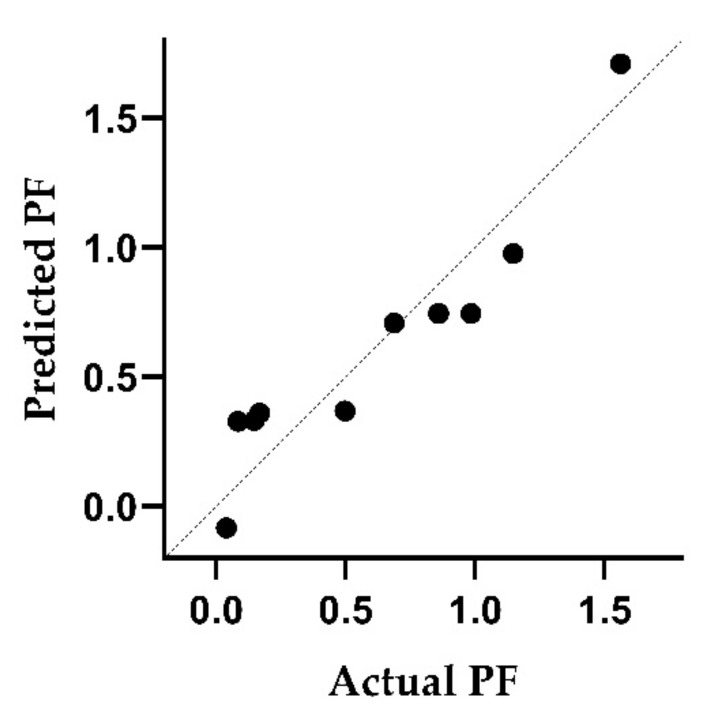
Predicted processing factor (PF) for pesticides in white wines in correlation with measured values. For the multiple linear regression, the log K_OW_ was taken into account and whether fining was carried out during winemaking.

The adjusted coefficient of determination indicated that the multiple linear regression model explains 85% of the variability in the PFs for white wine. An analysis of variance (ANOVA) showed that clarification has a significant effect (*p* < 0.05). Figure 3 illustrates the comparison of the experimental PFs with the predicted values obtained when applying Equation (2). The influence of the use of pectolytic enzymes was also investigated, but no significant effect was observed.

The overall results for white wine, showing a reduction in pesticide residues during winemaking, are consistent with the findings already described by other authors [9,10,12]. The correlation between the PF and the log K_OW_ of the active substance described in the literature for spiked samples [15] could be confirmed for incurred samples. This correlation is reasonable, given that in winemaking the extraction/separation of the juice from the solid parts of the grapes is of key importance and the partitioning of a pesticide between solid and liquid phases depends on its physicochemical properties. However, the present study revealed that, in addition to physicochemical properties, processing sub-steps, such as fining, must also be included in the calculation models in order to optimize the prediction.

Although it has been shown in various studies that different fining agents can have a pronounced and differing effect on the PF [13,16,30], the fining agent used was irrelevant for the calculation result. Nevertheless, it should be noted that only two different types of fining agents were used in the studies examined.

### 3.2. Red Wine

The results for the red wines are shown in Table 3. A median PF was estimated for 19 different pesticides. Similar to the white wines, the PF for all pesticides, with the exception of imidacloprid, was <1, suggesting that the pesticide concentrations generally decrease during winemaking. With a PF of 1 for imidacloprid in red wine, no change of concentration was observed during winemaking, while the corresponding PF for white wine of 1.57 indicated an increase. This difference could be due to the fact that the white wines in the imidacloprid studies were not fined. The treatment with fining agents and thus, the removal of solids, may have removed some imidacloprid in the red wine.

**Figure 4:**
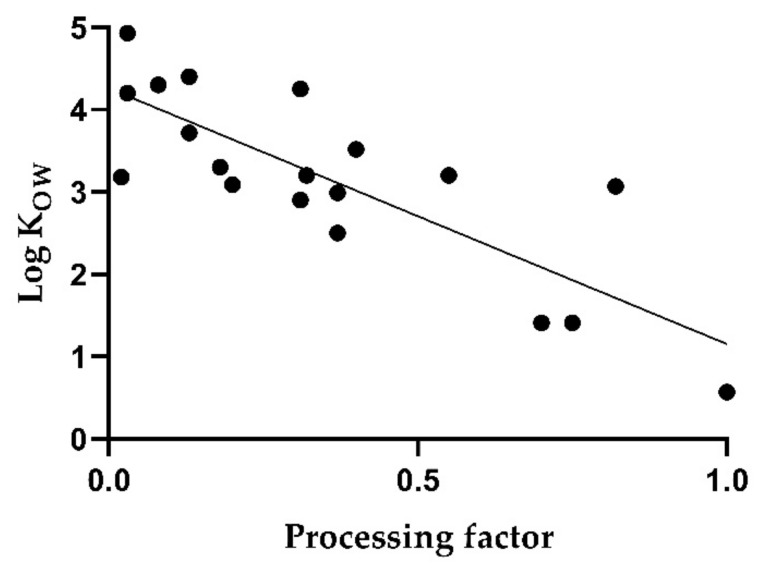
Correlation of median processing factors with octanol-water partition coefficients of pesticide active substances for red wines. Each dot represents one of the pesticides in Table 3.

Figure 4 shows a simple linear regression between the log K_OW_ and the median PF in red wine. The correlation is low with a coefficient of determination of 0.66 (*p* < 0.005). In particular, low log K_OW_ values, as for imidacloprid and thiophanate methyl, are associated with higher PFs. On the other hand, valifenalate exhibits a relatively high processing factor of 0.82 despite a log K_OW_ of 3.07.

The low correlation is attributed to additional influencing factors resulting from the different processing techniques, which may interfere with the PF.

**Figure 5:**
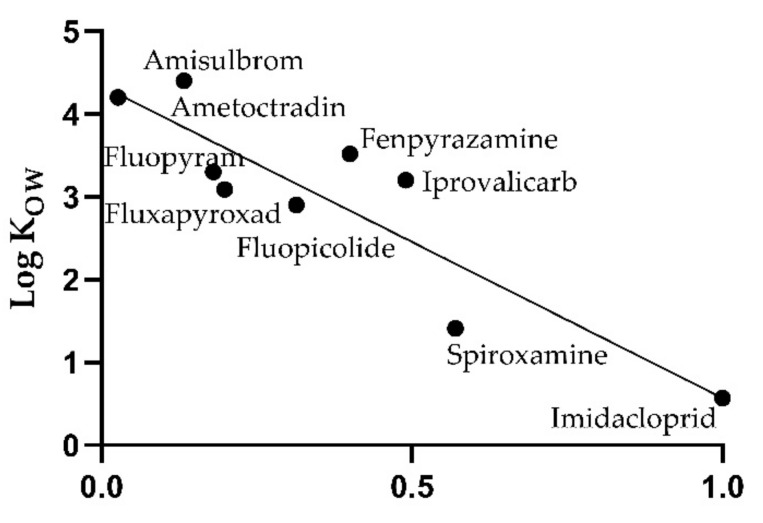
Correlation of median processing factors with octanol-water partition coefficients of pesticides for red wines produced by thermovinification.

One of the main differences between the various processing methods was whether mash fermentation or thermovinification was applied. When evaluating the correlation between the log K_OW_ and the PF separately for both methods of red wine production, a clear correlation was shown for thermovinification (Figure 5), resulting in a coefficient of determination of 0.81 (*p* < 0.005). The equation of the linear regression is as follows:

(3) $$\mathrm{PF}=1.151-0.23{\;\mathrm{logK}}_{\mathrm{OW}}$$

For red wine produced with mash fermentation, only a weak correlation with a coefficient of determination of 0.63 (*p* < 0.005) was determined.

For the pesticide spiroxamine, sufficient data were available for both methods, the thermovinification and the classical mash fermentation. A comparison of the median PF clearly shows that with thermovinification (PF = 0.57) less spiroxamine residues were transferred into the wine than with mash fermentation (PF = 0.96). A similar behavior was observed in the study by Liapis et al. [31], where mash fermentation resulted in higher spiroxamine residues in wine compared to must fermentation. However, since only one pesticide was tested, employing both methods, no general statement can be made about which method leads to higher residues.

The fact that the PFs for red wine produced with thermovinification correlate better with the log K_OW_ values than the PFs obtained from red wine produced with mash fermentation could be due to the different time points at which the solids are separated from the liquid in the two different production processes.

In red wine production with thermovinification, the pomace is separated from the must before fermentation. A large part of the pesticides often accumulates in the pomace and is therefore also removed in this step. This is especially relevant for pesticides with low log K_OW_ values. In red wine production with mash fermentation, solids are separated after the fermentation. The pesticides bound to the grape matrix can get in contact with the developed ethanol during the fermentation process. For this reason, the solubility of the pesticides in ethanol could have an additional influence on the PF. Due to the lack of data on the solubility of the investigated pesticides in ethanol, this hypothesis could not be verified within the scope of this work.

An influence of ethanol solubility on PFs is also discussed in the study by Fernandez-Alba et al. [15]. In this study, it was found that pesticides with good ethanol solubility are more stable during fermentation compared to pesticides with similar log K_OW_ but lower ethanol solubility.

The thermovinification process and the production of white wine are very similar. The main difference is that in the thermovinification of red wine, unlike white wine, the mash is heated before pressing. It is assumed that this affects only thermolabile pesticides. All of the active ingredients examined are stable at temperatures between 60–80 °C. When the results obtained with both methods are evaluated together in a multiple linear regression, there is also a correlation between the log K_OW_ and the PF (adjusted R^2^ = 0.81; *p* < 0.005). An analysis of variance (ANOVA) showed that clarification has a significant effect (*p* < 0.005). However, whether it is white wine or red wine produced by thermovinification has no significant influence (*p* = 0.73). These results suggest that processing factors can be extrapolated from one process to the other. Further data are needed to confirm this thesis. Another factor that could influence the processing factor is the grape variety. This is because the composition, as well as the structure of the skin and the pulp, differ greatly between the varieties. However, due to the high number of varieties used in the studies examined, this thesis could not be investigated within the framework of this work.

## 4. Conclusions

In the present study, it was shown that the transfer of incurred pesticide residues from grapes into wine during winemaking correlates with the logarithmic octanol-water coefficients of the substances under certain processing conditions. For white wines, a multiple linear regression method was useful for investigating the different parameters that potentially affect pesticide residues during winemaking. A pronounced multiple linear correlation of the PF was found with the log K_OW_ and the treatment with fining agents.

For red wine, a correlation of the log K_OW_ with the PF was determined as well. When looking at the two different production methods used in the studies individually, red wine produced by thermovinification showed a stronger correlation. As the mash fermentation process involves contact of pesticides with the developing ethanol, it is assumed that their solubility in ethanol has an additional effect on the residue transfer. However, this hypothesis could not be further investigated within the scope of this work.

As the investigated processing studies represent only a small selection of winemaking conditions, it can be assumed that the variation of PFs in real samples is even higher. This should be taken into account when interpreting and applying a PF. The present study suggests that the processing factors for white wine and for red wine produced with the thermovinification method are comparable. Further studies to confirm this correlation would improve the prediction of PFs.

These first results indicate that prediction models for processing factors can be developed based on physicochemical properties of pesticide-active substances, when sufficient details about the food processing are known. Detailed information on certain process parameters should be made mandatory for studies conducted according to OECD Guideline 508. For example, it is crucial to know whether fining was part of the process and if so, which, and how much of a certain fining agent was used. Validated prediction models can be useful tools to describe the likely behavior of a pesticide during food processing, when no experimental evidence is available. The authors would like to encourage further work in this area, namely the in-depth evaluation of further processes and the establishment of respective prediction models.

As the database is still limited, even for the well-investigated process of winemaking, it is recommended to continue validating the prediction model with winemaking studies becoming available for further pesticides in future.

## Figures and Tables

**Table 1:** Logarithm of the octanol-water partition coefficient (log K_OW_) and water solubility (S_W_, at 20 °C) of the investigated pesticides.

Pesticide	Function	log K_OW_	S_W_ [mg L^−1^]
Ametoctradin	Fungicide	4.20	0.23
Amisulbrom	Fungicide	4.40	0.11
Azoxystrobin	Fungicide	2.50	6.70
Benzovindiflupyr	Fungicide	4.30	0.98
Fenpyrazamine	Fungicide	3.52	20.4
Fluopicolide	Fungicide	2.90	2.80
Fluopyram	Fungicide	3.30	16.0
Fluxapyroxad	Fungicide	3.09	3.78
Imidacloprid	Insecticide	0.57	610
Iprodione	Fungicide	2.99	8.90
Iprovalicarb	Fungicide	3.20	17.8
Mandipropamid	Fungicide	3.20	4.20
Mepanipyrim	Fungicide	3.18	4.60
Myclobutanil	Fungicide	3.17	124
Penconazole	Fungicide	3.72	73.0
Spiroxamine	Fungicide	1.41	340
Tebufenozide	Insecticide	4.25	0.83
Tebufenpyrad	Insecticide	4.93	3.20
Thiophanate-methyl	Fungicide	1.41	22.4
Valifenalate	Fungicide	3.07	24.1

**Table 2:** Median processing factors (PF) for different pesticides in clarified (+) and non-clarified (−) white wine.

Pesticide	Clarification	PF Must	PF Wine	No. of PFs
Benzovindiflupyr	+	0.57	0.03	4
Fluopyram	−	0.88	0.69	2
Imidacloprid	−	1.86	1.57	2
Iprovalicarb	−	0.72	0.86	2
Mandipropamid	−	-	0.99	2
Mepanipyrim	+	0.59	0.09	4
Myclobutanil	+	0.22	0.15	3
Tebufenozide	−	0.12	0.17	5
Thiophanate-methyl	+	-	1.15	3
Valifenalate	+	0.73	0.50	5

**Table 3:** Median processing factor (PF) for different pesticides in red wine produced with (+) and without (−) thermovinification.

Pesticide	Thermovinification	PF	No. of PFs
Ametoctradin	+	0.03	4
Amisulbrom	+	0.13	3
Azoxystrobin	−	0.37	6
Benzovindiflupyr	−	0.08	4
Fenpyrazamine	+	0.40	3
Fluopicolide	+	0.31	3
Fluopyram	+	0.18	4
Fluxapyroxad	+	0.20	2
Imidacloprid	+	1.00	3
Iprodione	−	0.37	5
Iprovalicarb	+	0.55	2
Mandipropamid	−	0.32	2
Mepanipyrim	−	0.02	3
Penconazole	−	0.13	3
Spiroxamine	−	0.96	2
Spiroxamine	+	0.57	3
Tebufenozide	−	0.31	5
Tebufenpyrad	−	0.03	2
Thiophanate-methyl	−	0.75	3
Valifenalate	−	0.82	4

## Data Availability

Publicly available datasets were analyzed in this study. This data can be found here: https://doi.org/10.5281/zenodo.1488653 (accessed on 4 April 2022).
